# Supplementary material for: Modulating supramolecular binding of carbon dioxide in a redox-active porous metal-organic framework
Source: Nat Commun. 2017 Feb 13;8:14212. doi: 10.1038/ncomms14212 (PMC5316804; doi:10.1038/ncomms14212)
Supplement: Supplementary Information — Supplementary Figures, Supplementary Tables, Supplementary Methods and Supplementary References [file ncomms14212-s1.pdf]

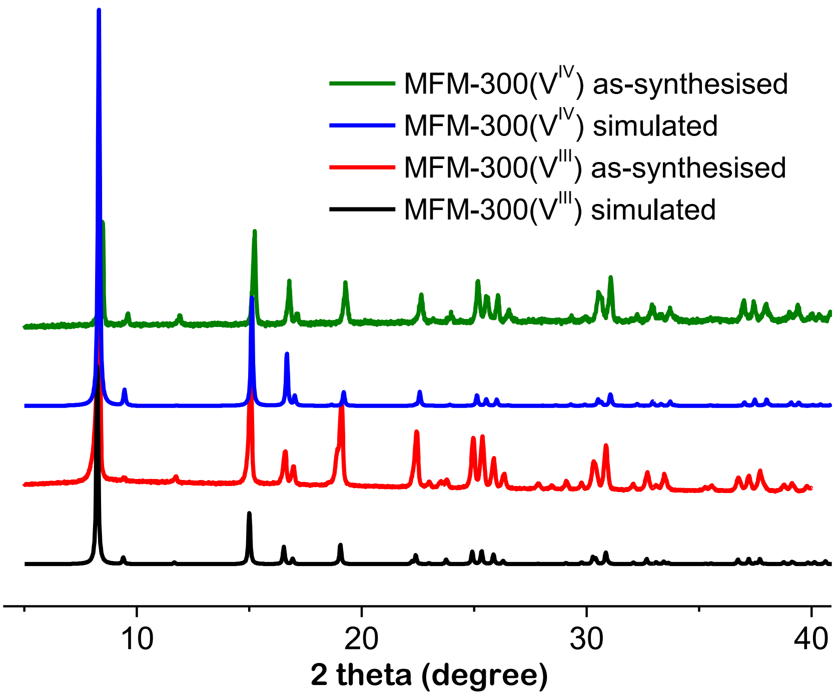

Supplementary Figure 1. Powder X-ray diffraction patterns for as-synthesised MFM-300(V^{III}) and MFM-300(V^{IV}).

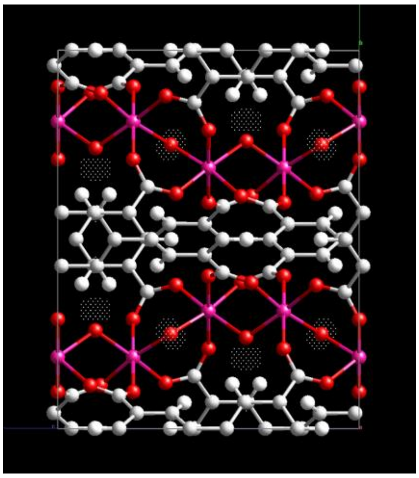

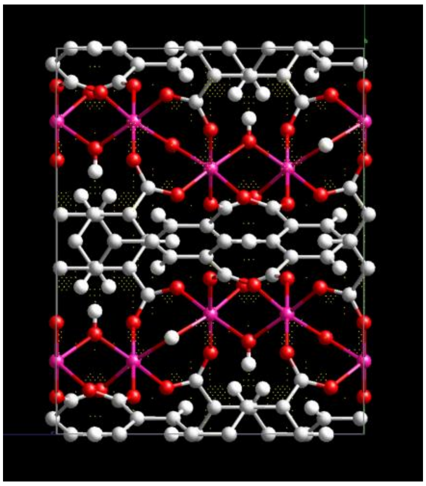

a b

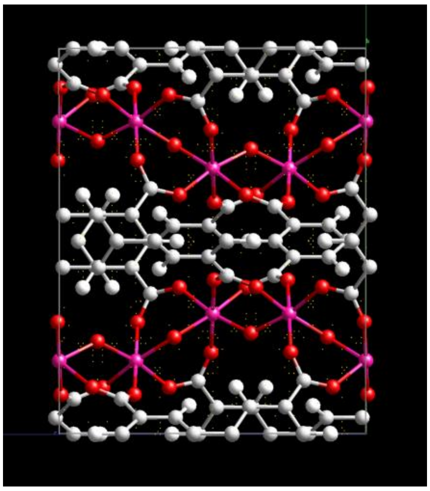

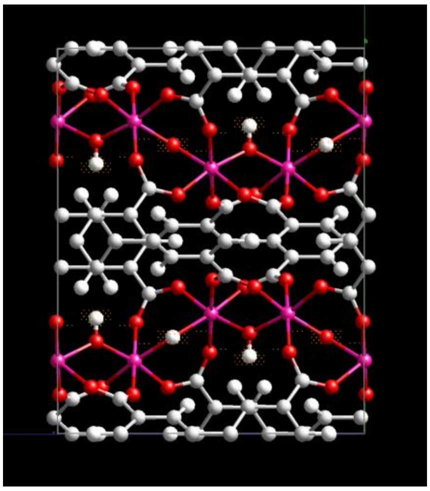

c d

Supplementary Figure 2. Comparison of NPD difference Fourier maps after adding or omitting the hydrogen atom on the bridging oxygen atom within MFM-300(V^{III}) or MFM-300(V^{IV}). (a) For MFM-300(V^{III}), refinement after removal of H on the bridging O shows negative residual density peaks. (b) For MFM-300(V^{III}), refinement after addition of H on the bridging O atom confirms no evident density peaks indicative of the presence of the hydroxyl group. (c) For MFM-300(V^{IV}), refinement after removal of H on the O^{2-} bridges confirms no evident density peaks indicative of deprotonation of the O^{2-} bridge. (d) For MFM-300(V^{IV}), refinement after addition of H on the bridging O^{2-} atom shows positive density peaks (light grey, carbon; white, hydrogen; red, oxygen; purple, vanadium; dotted area, density peaks).

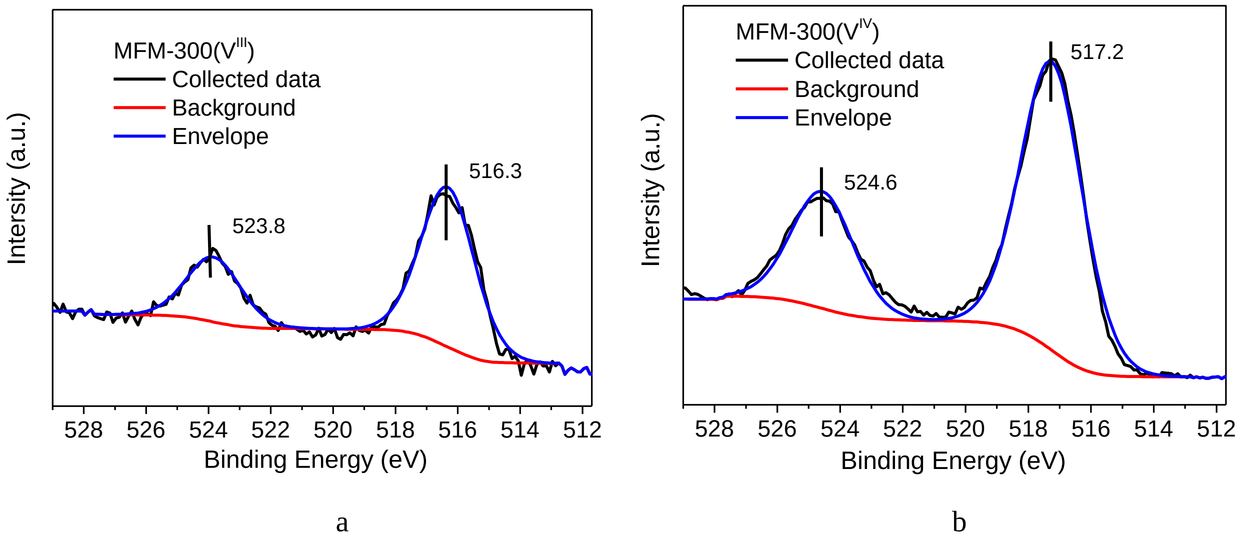

Supplementary Figure 3. XPS spectra of desolvated (a) MFM-300(V^{III}) and (b) MFM-300(V^{IV}). The fitted curves are shown in blue.

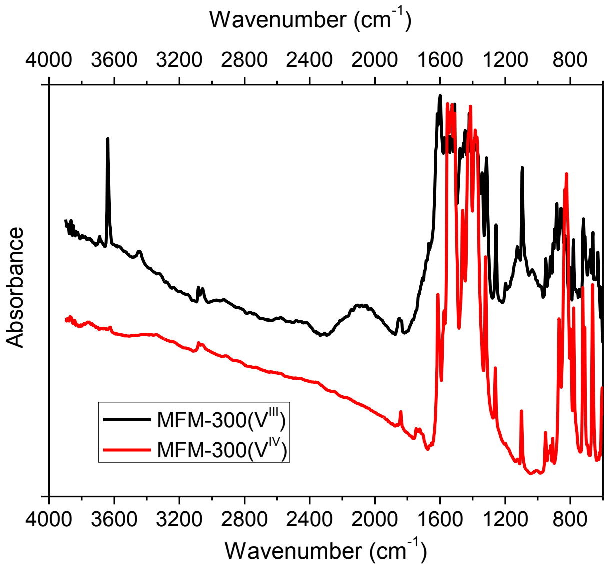

Supplementary Figure 4. Comparison of the synchrotron micro-IR spectra of desolvated MFM-300(V^{III}) and MFM-300(V^{IV}).

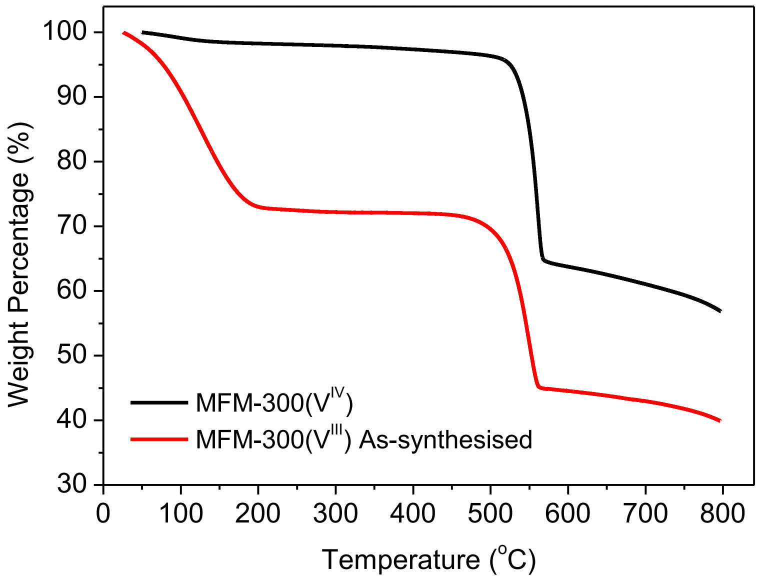

Supplementary Figure 5. TGA plots for MFM-300(V^{III}) and MFM-300(V^{IV}) under N_2 atmosphere.

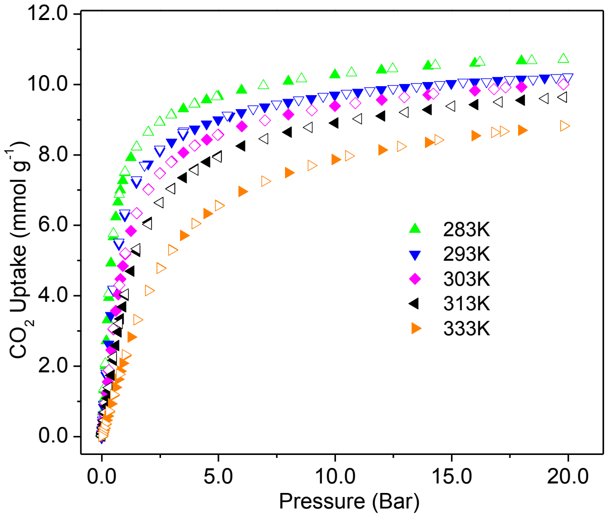

a

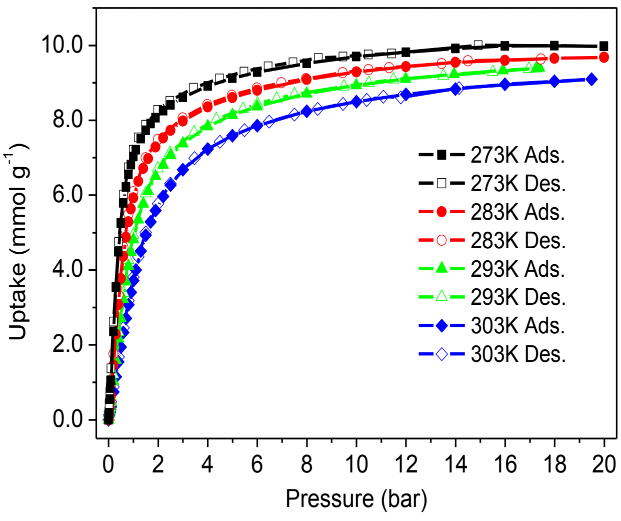

b

Supplementary Figure 6. CO_2 isotherms for (a) MFM-300(V^{III}) and (b) MFM-300(V^{IV}).

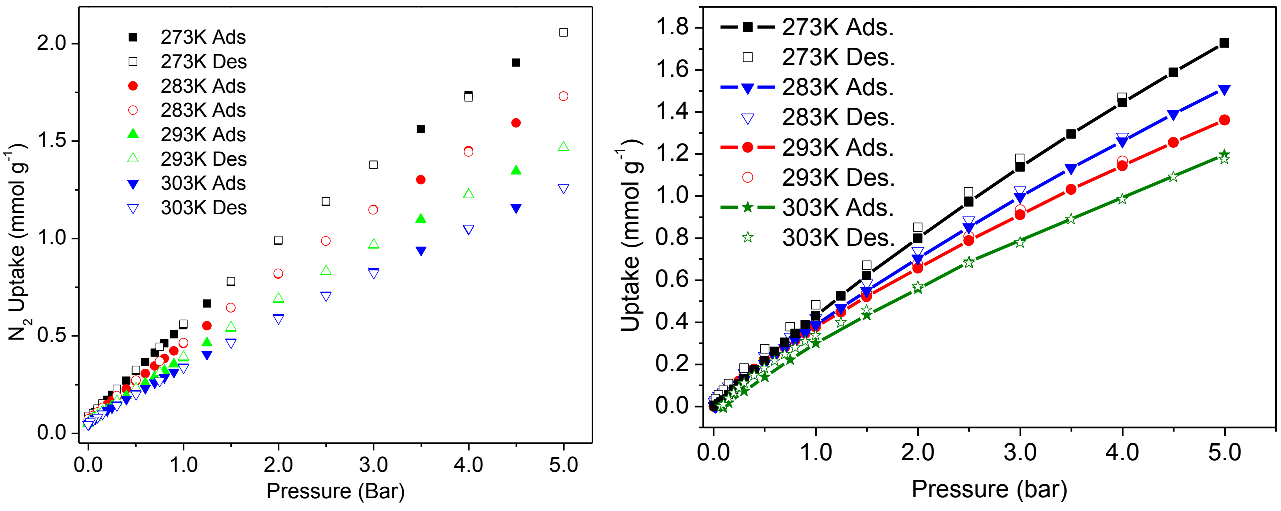

a b

Supplementary Figure 7. N_2 isotherms for (a) MFM-300(V^{III}) and (b) MFM-300(V^{IV}).

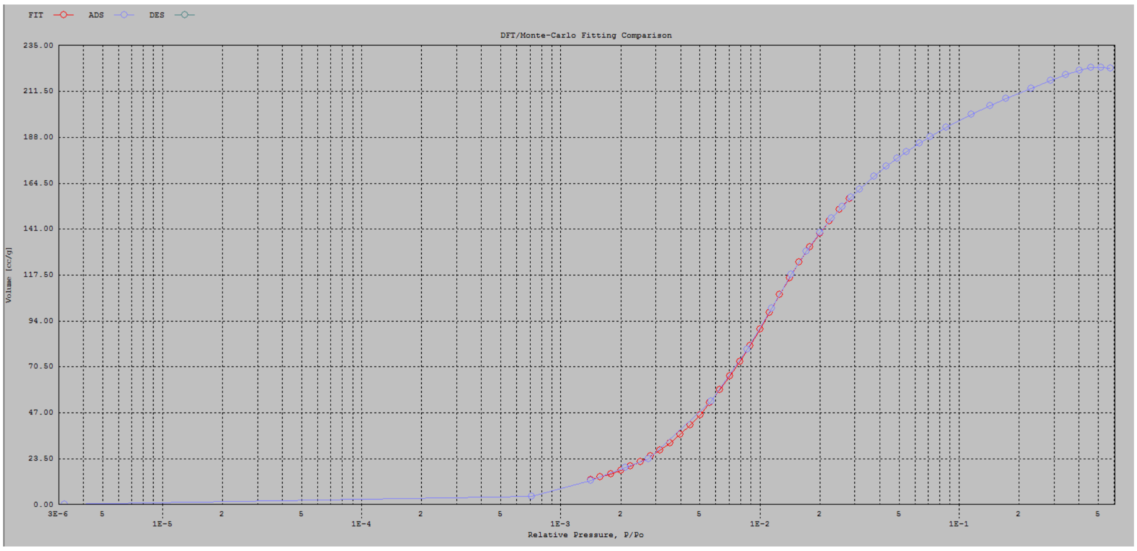

Supplementary Figure 8. DFT/Monte-Carlo fitting for CO_2 isotherm of MFM-300(V^{IV}) at 273K, giving a surface area of 1565 m^2 g^{-1} and pore volume of 0.489 cm^3 g^{-1}.

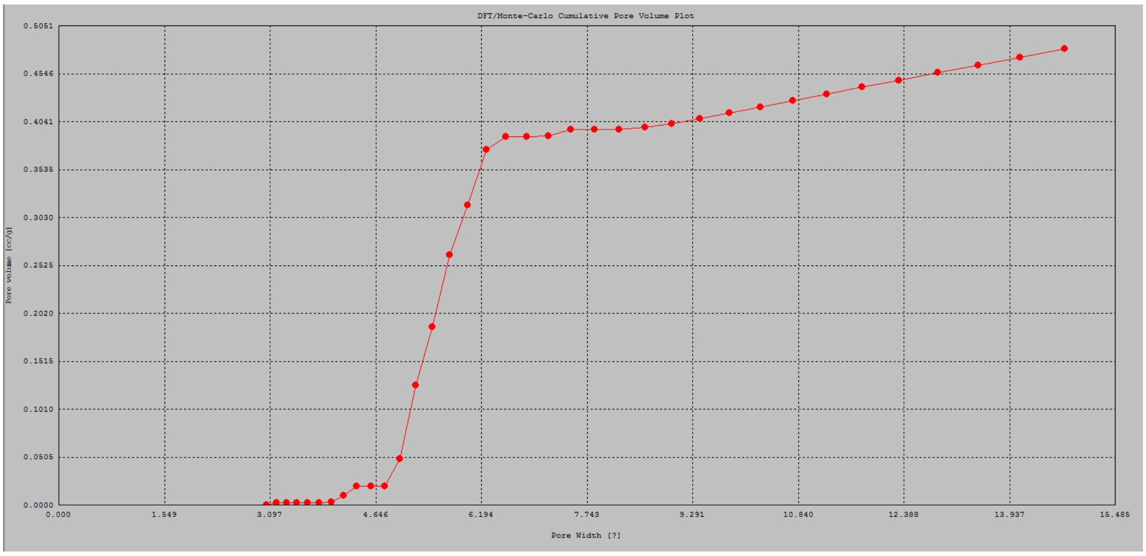

Supplementary Figure 9. DFT/Monte-Carlo cumulative pore volume plot for MFM-300(V^{IV}).

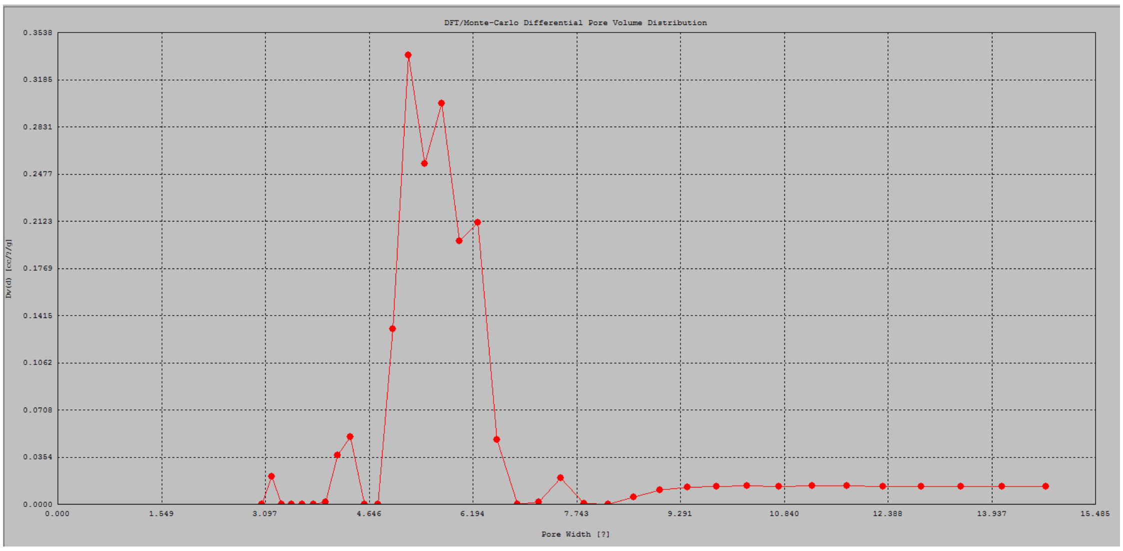

Supplementary Figure 10. DFT/Monte-Carlo differential pore volume distribution plot for MFM-300(V^{IV}), giving a pore size of 5.5 Å.

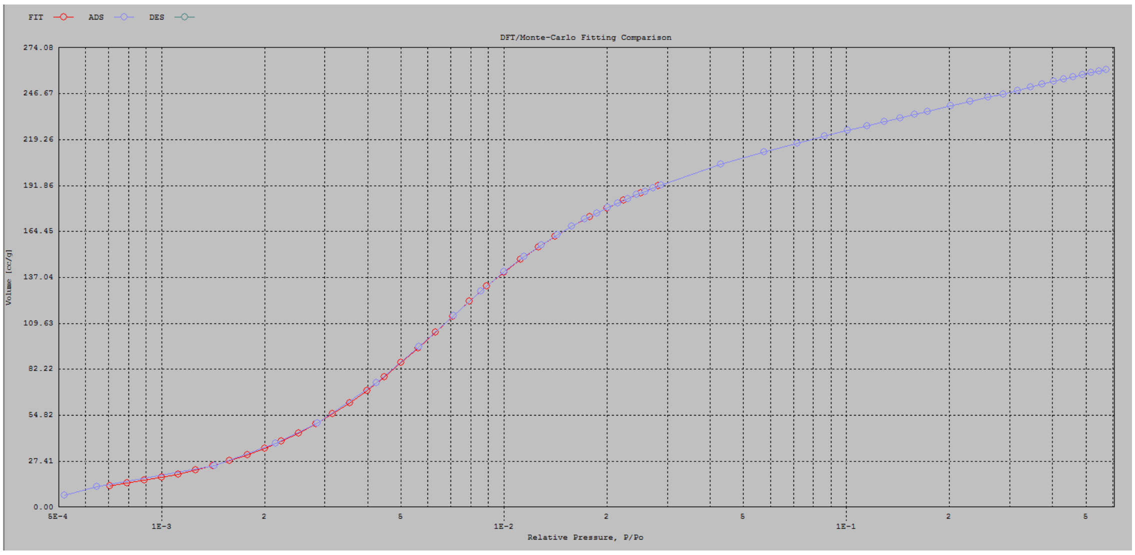

Supplementary Figure 11. DFT/Monte-Carlo fitting for CO_2 isotherm of MFM-300(V^{III}) at 273K, giving a surface area of 1892 m^2 g^{-1} and pore volume of 0.490 cm^3 g^{-1}.

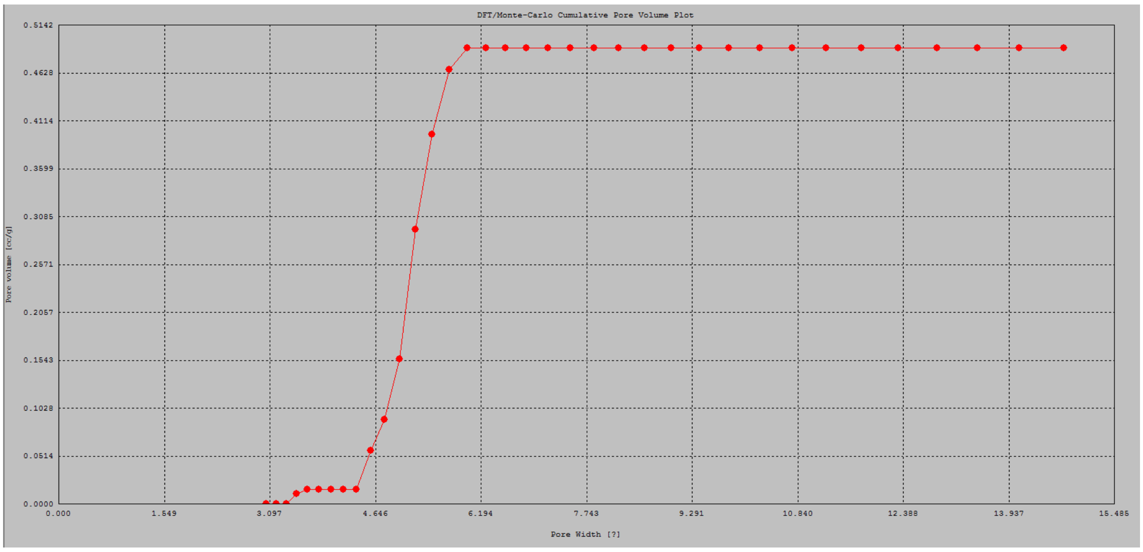

Supplementary Figure 12. DFT/Monte-Carlo cumulative pore volume plot for MFM-300(V^{III}).

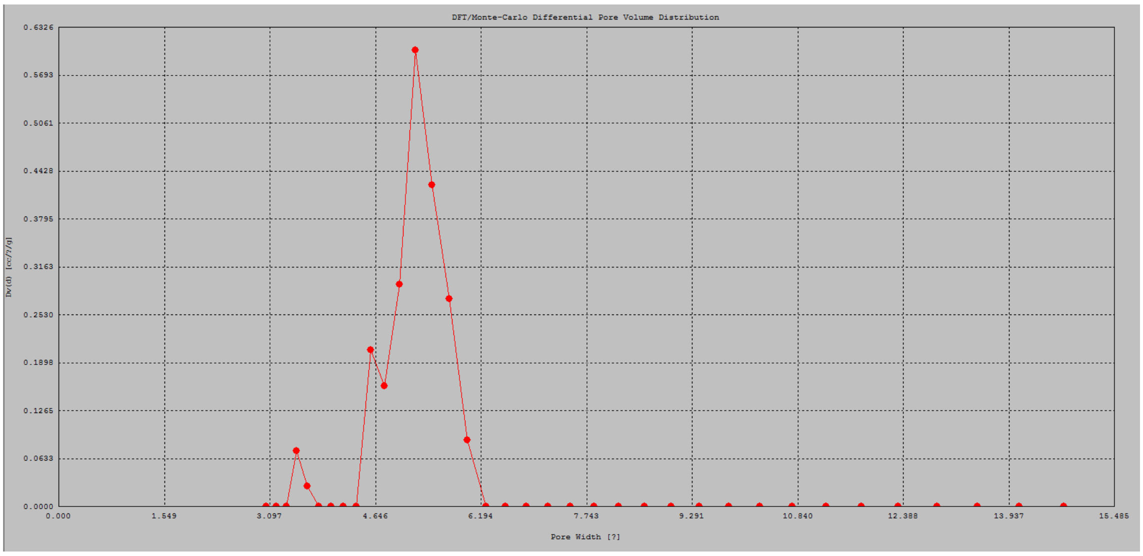

Supplementary Figure 13. DFT/Monte-Carlo differential pore volume distribution plot for MFM-300(V^{III}), giving a pore size of 5.24 Å.

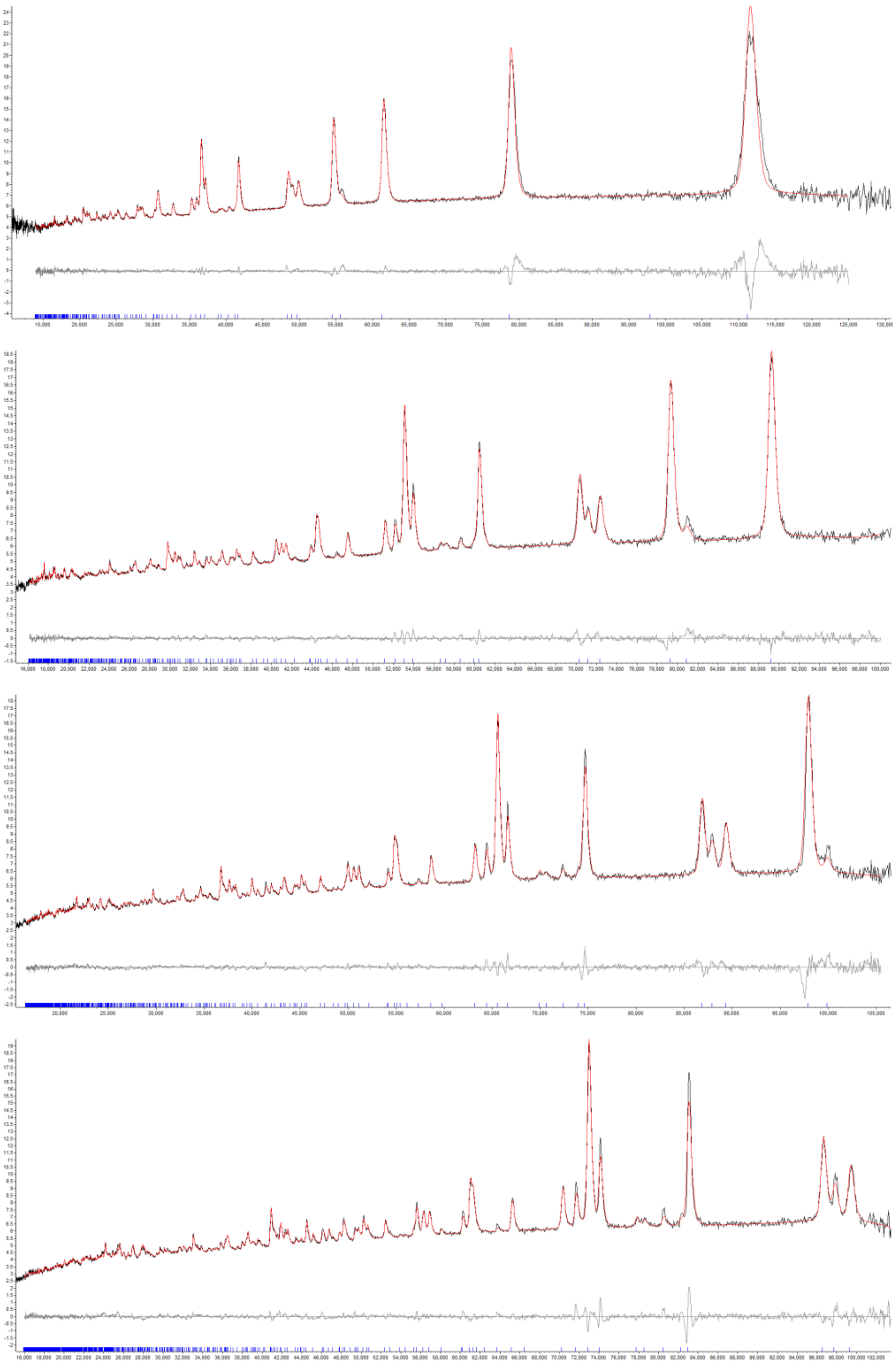

Supplementary Figure 14. Neutron diffraction patterns and Rietveld refinement for 1.4CO_2@MFM-300(V^{III}) (banks 2 to 5 from top to the bottom).

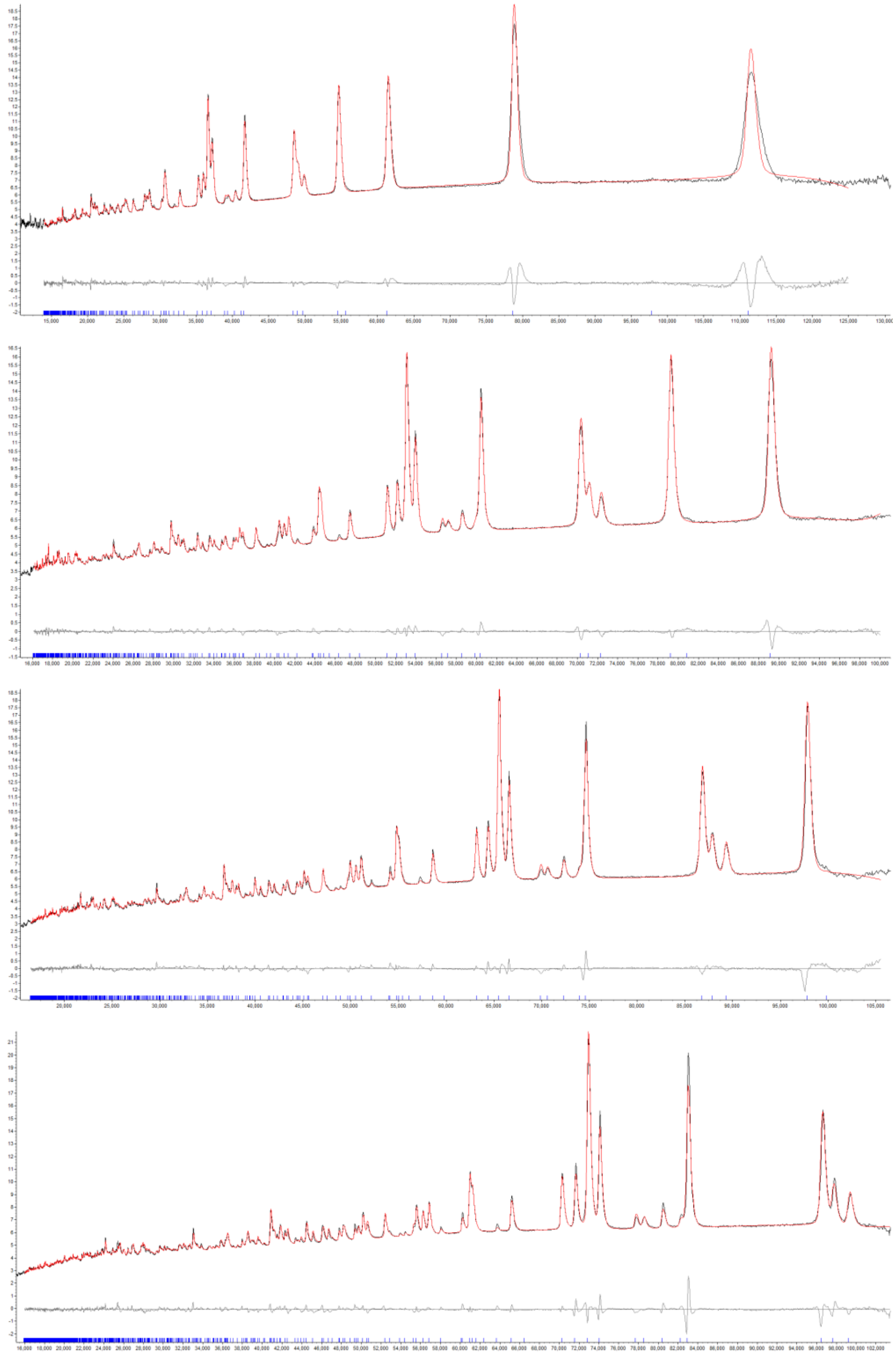

Supplementary Figure 15. Neutron diffraction patterns and Rietveld refinement for $2.0CO_2$@MFM-300(V^{III}) (banks 2 to 5 from top to the bottom).

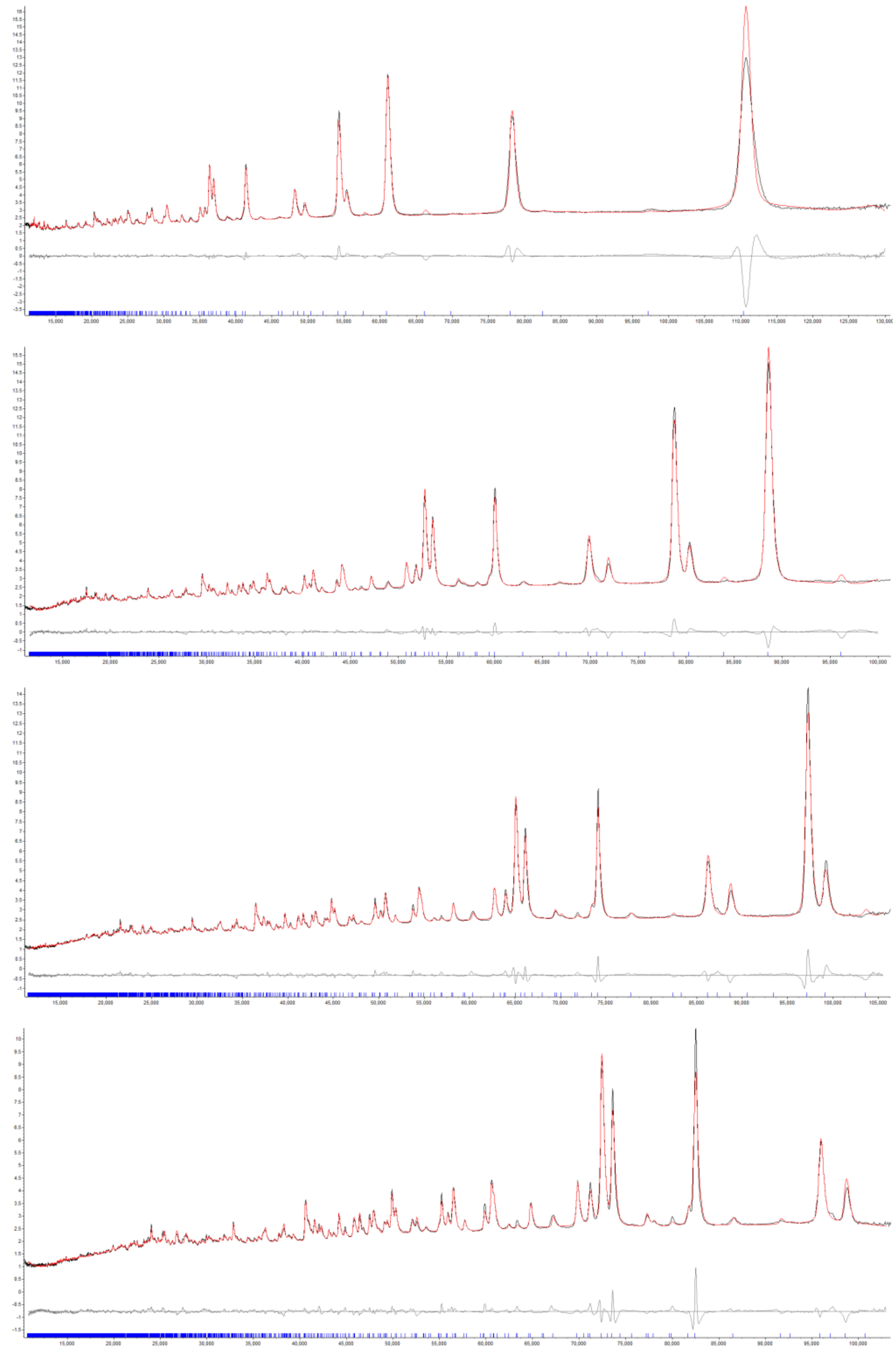

Supplementary Figure 16. Neutron diffraction patterns and Rietveld refinement for 1.4CO_2@MFM-300(V^{IV}) (banks 2 to 5 from top to the bottom).

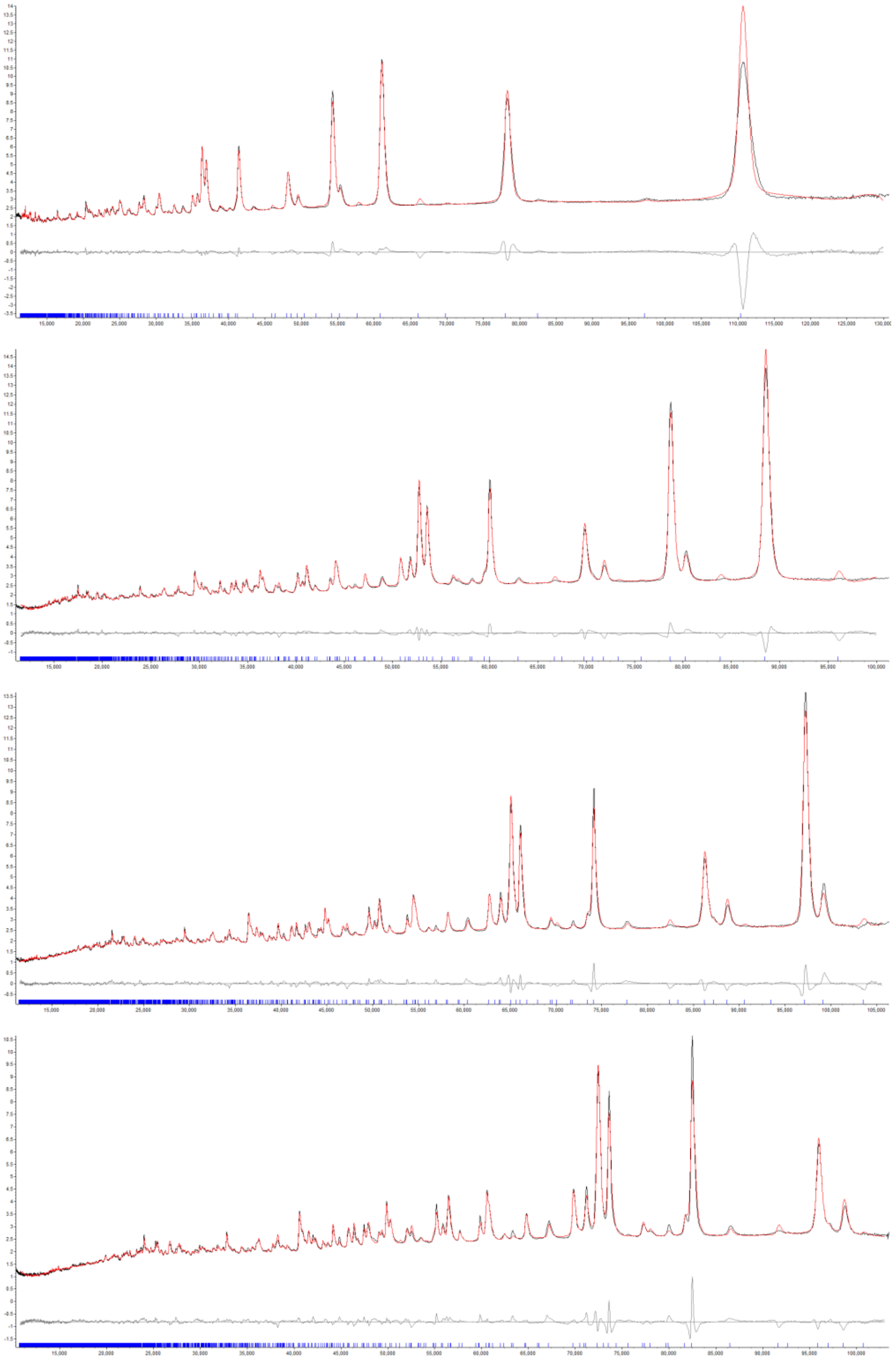

Supplementary Figure 17. Neutron diffraction patterns and Rietveld refinement for $1.6CO_2$@ MFM-300(V^{IV}) (banks 2 to 5 from top to the bottom).

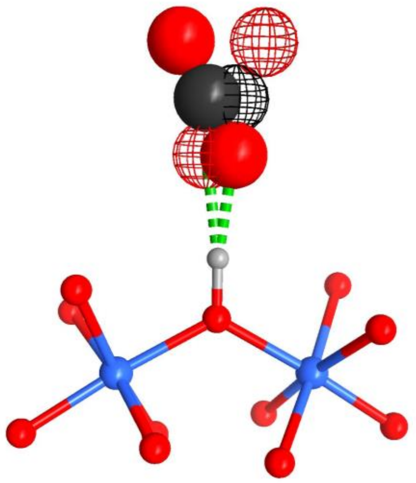

Supplementary Figure 18. Diagram showing the disordered CO_2 on site I interacting with the hydroxyl group within CO_2-loaded MFM-300(V^{III}).

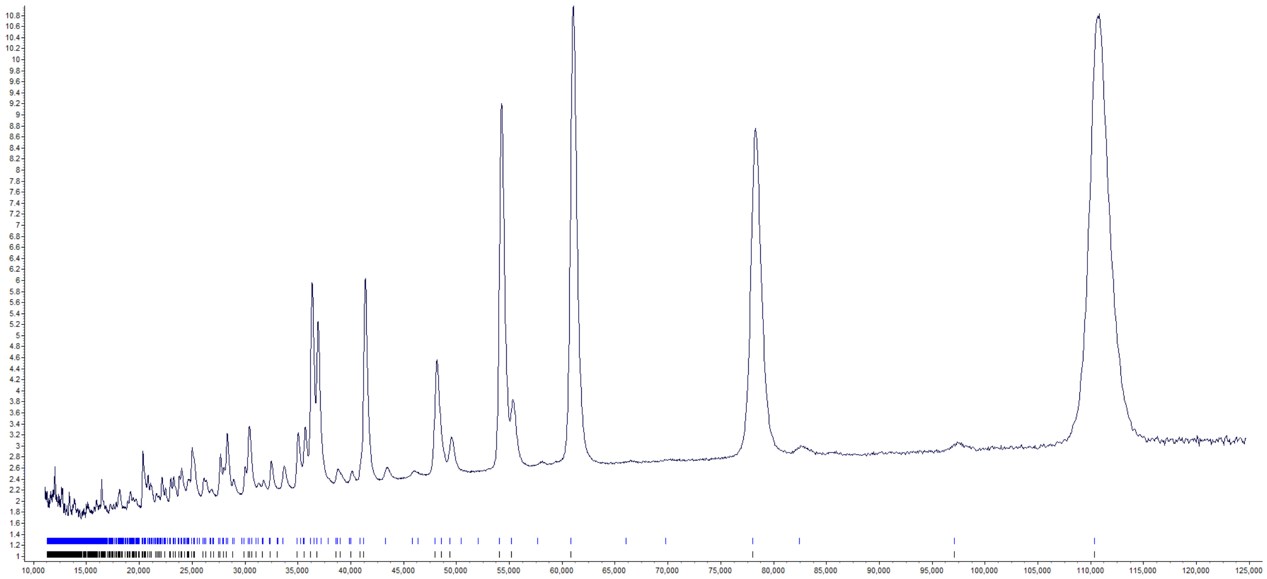

Supplementary Figure 19. Observed NPD pattern for 1.6CO_2@MFM-300(V^{IV}) (bank 2 on WISH diffractometer at ISIS). Tick marks at the bottom of the image show allowed Bragg reflection positions for space groups $P4_122$ and $I4_122$ (blue and black, respectively).

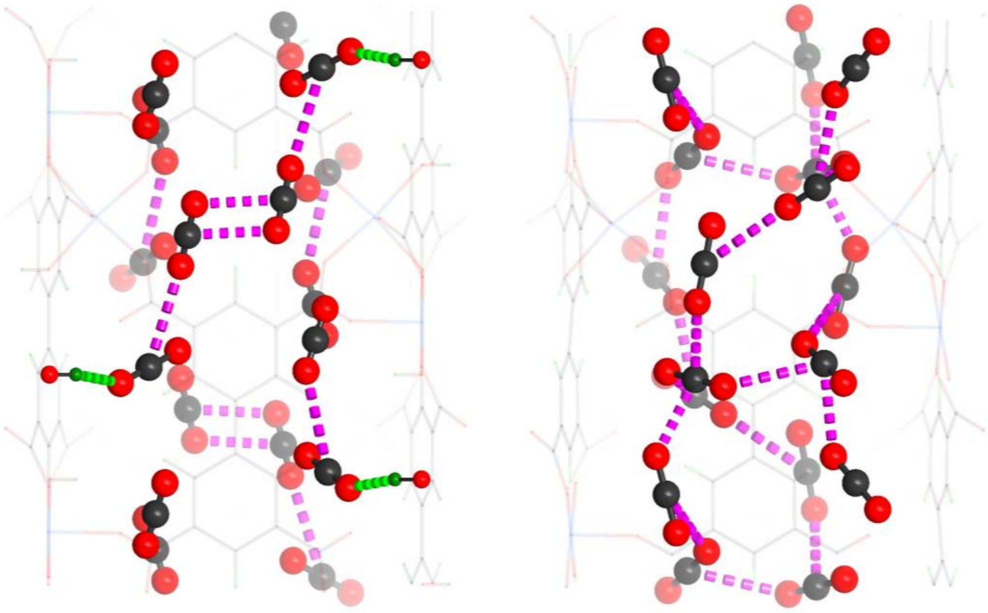

a b

Supplementary Figure 20. Side view showing the positions of CO_2 in (a) MFM-300(V^{III}) and (b) MFM-300(V^{IV}) at a loading of 1.4 CO_2/V.

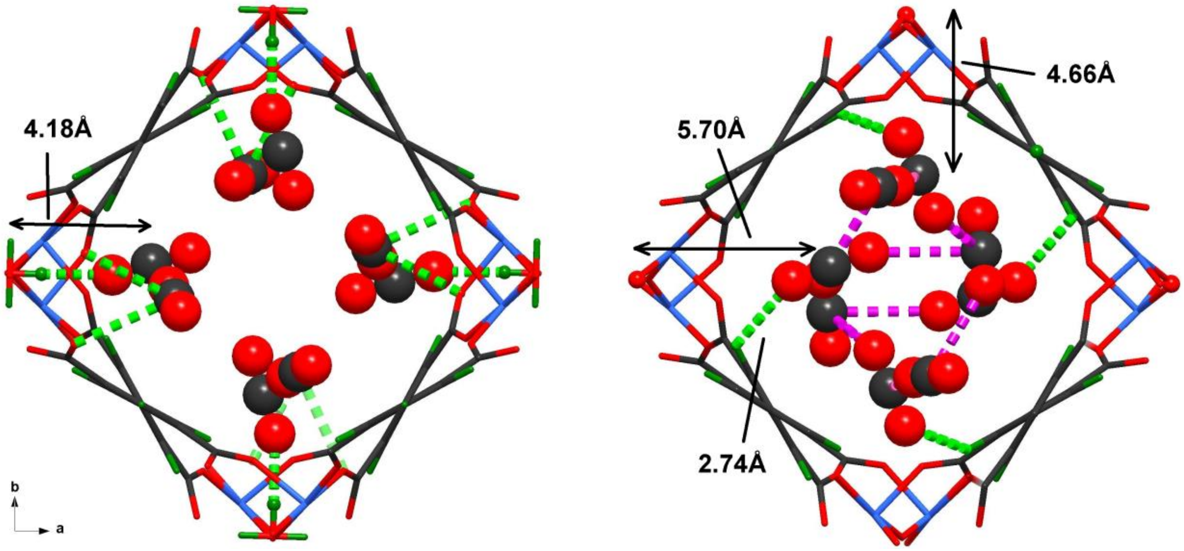

a b

Supplementary Figure 21. Diagrams showing the packing and interaction of adsorbed CO_2 within (a) MFM-300(V^{III}) and (b) MFM-300(V^{IV}). Data were obtained by DFT simulation.

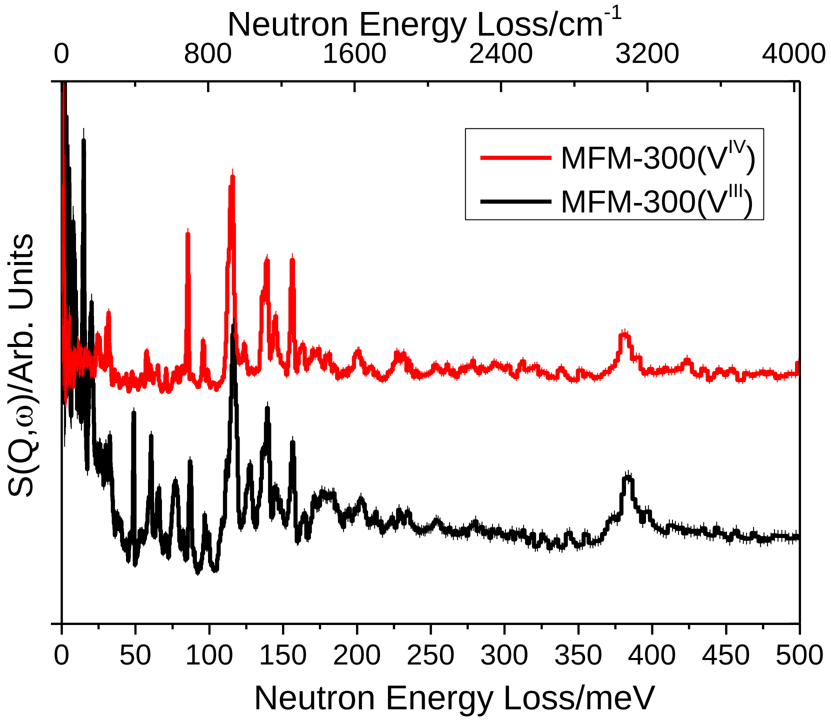

Supplementary Figure 22. Comparison of the INS spectra for MFM-300(V^{III}) and MFM-300(V^{IV}).

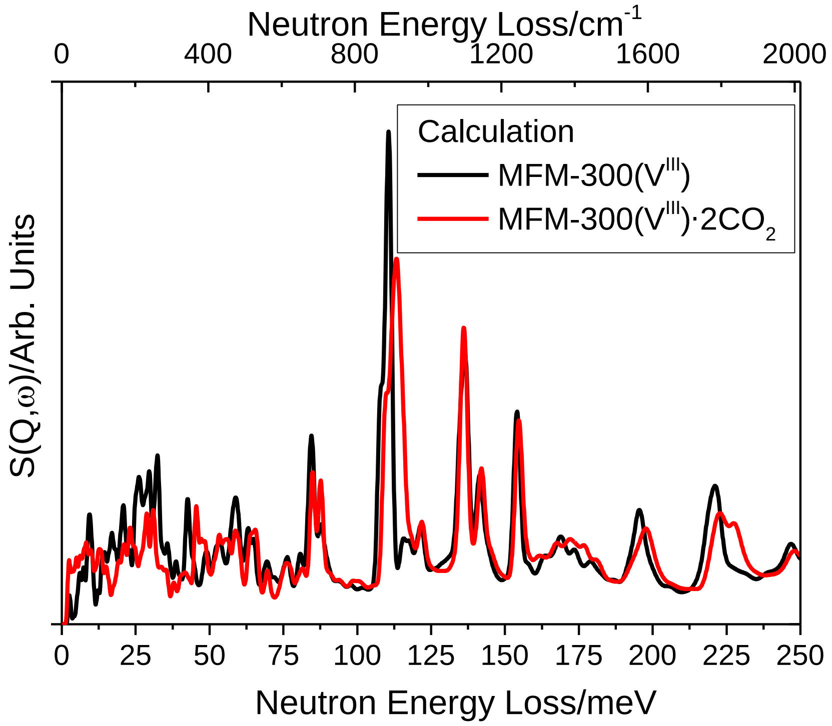

Supplementary Figure 23. Comparison of the calculated INS spectra for bare and CO_2-loaded MFM-300(V^{III}).

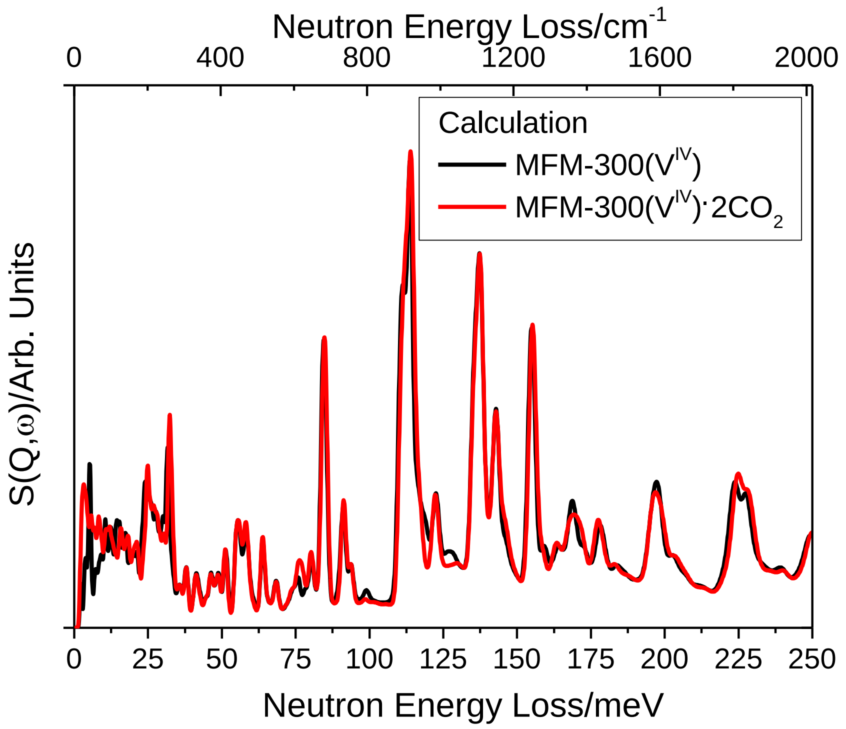

Supplementary Figure 24. Comparison of the calculated INS spectra for bare and CO_2-loaded MFM-300(V^{IV}).

Supplementary Table 1. Single Crystal Data for MFM-300(V).

	MFM-300(V^{III})	MFM-300(V^{IV})
Formula	$C_{16}H_8O_{10}V_2$	$C_{16}H_6O_{10}V_2$
MoKa,	0.7749	0.7749
Crystal system	Tetragonal	Tetragonal
Mr	462.1	460.1
Space group	$I\,4_122$	$I\,4_122$
a, *b* (Å)	15.117(1)	15.024(1)
c (Å)	12.083(1)	11.936(1)
Volume (Å^3)	2761.4(5)	2694.2(4)
Z	4	4
ρ_{calc} Mg/m^3	1.112	1.134
μ, mm^{-1}	0.712	0.729
F(000)	920	912
Refl. collected	5274	6943
Independent refl.	2696	1736
Final R indices(R1)	0.0387	0.0594
[I>2σ(I)] wR2	0.0747	0.0886
(all data) wR2	0.0771	0.0935
GOF	1.050	1.054
CCDC	1479680	1479681

Supplementary Table 2. Crystal Data and Details of the Structure Determination for: CO_2@MFM-300(V)

	1.4CO_2@MFM-300(V^{III})	2.0CO_2@MFM-300(V^{III})	1.4CO_2@MFM-300(V^{IV})	1.6CO_2@MFM-300(V^{IV})
Formula	$C_8H_4O_5V·1.41(CO_2)$	$C_8H_4O_5V·2.04(CO_2)$	$C_8H_3O_5V·1.38\ (CO_2)$	$C_8H_3O_5V·1.59(CO_2)$
Formula Weight	293.02	320.74	290.46	300.07
Crystal System	Tetragonal	Tetragonal	Tetragonal	Tetragonal
Space group	$I4_122$	$I4_122$	$P4_122$	$P4_122$
a, b [Angstrom]	15.1658(2)	15.16059(14)	15.04185(12)	15.03934(12)
c [Angstrom]	12.0568(3)	12.0474(2)	11.96113(19)	11.9587(2)
V [Ang^3]	2773.08(9)	2769.02(6)	2706.29(5)	2704.84(5)
Z	8	8	8	8
D(calc) [g/cm^3]	1.404	1.539	1.426	1.474
F(000)	1168	1279	1154	1192
Radiation type	Neutron	Neutron	Neutron	Neutron
Scan method	Time of Flight	Time of Flight	Time of Flight	Time of Flight
R_{exp}	1.32	0.29	0.32	0.32
R_{wp}	1.98	1.73	2.31	2.51
R_p	1.92	1.40	2.13	2.21
GoF	1.49	5.85	7.31	7.89
CCDC	1481144	1481145	1481146	1481147

Supplementary Methods

Materials and other measurements.

All reagents were used as received from commercial suppliers without further purification. Analyses for C, H and N were carried out on a CE-440 elemental analyzer (EAI Company). Thermal gravimetric analyses (TGA) were performed under N_2 flow (100 ml/min) with a heating rate of 2 °C/min using a TASDT-600 thermogravimetric analyser (TA Company). Powder X-ray diffraction data (PXRD) were collected over the 2θ range 4-50° on a Bruker Advance D8 diffractometer using Cu-Kα_1 radiation (λ = 1.54056 Å, 40 kV/40mA). XPS spectra were collected on a Kratos Axis Ultra X-ray photoelectron spectrometer equipped with an aluminium/magnesium dual anode and a monochromated aluminium X-ray source.

Single crystal Data and Details of the Structure Determination for MFM-300(V)

Single crystal X-ray structures were solved by direct methods and refined by full matrix least-squares on F^2 using the SHELXL software package.[1] Non-hydrogen atoms were refined with anisotropic displacement parameters during the final cycles. Hydrogen atoms on the organic ligands were calculated in ideal positions with isotropic displacement parameters 1.2 times to those of parent carbon atoms. The hydrogen atom on the hydroxyl group was located from the electron difference map with isotropic displacement parameters 1.5 times to those of parent oxygen atom, and the O-H bond length was restrained to have a value of 0.85 Å. It is worth noting that usually in X-ray crystal structure analysis, it is very difficult to locate hydrogen atoms from the residual electron map. However, in this case, the positions of hydrogen atoms from the residual electron map can be readily identified. Moreover, the position of the hydrogen atoms has been further confirmed from neutron diffraction analysis.

Neutron Powder Diffraction (NPD) Experiments and Structure Determination.

Neutron Powder Diffraction experiments were carried out at WISH, a long wavelength powder and single crystal neutron diffractometer at the ISIS Facility at the Rutherford Appleton Laboratory (UK). The instrument views a solid methane moderator providing a high flux of cold neutrons with a large bandwidth, transported to the sample *via* an elliptical guide. The WISH divergent jaws system allows for tuning the resolution according to the need of the experiment; in this case, it was setup in high resolution mode. The WISH detectors are 1m long, 8mm diameter pixellated 3He tubes positioned at 2.2m from the sample and arranged on a cylindrical locus covering 10-170 degrees in 2 theta scattering angle. To reduce the background from sample environment, it is equipped with an oscillating radial collimator that defines a cylinder of radius about 22mm diameter at 90 degrees scattering.

The sample of desolvated MFM-300(V^{III}) was loaded into a cylindrical vanadium sample container with an indium vacuum seal and connected to a gas handling system. The sample was degassed at 10^{-7} mbar and 100 °C for 1 day to remove any remaining trace guest water molecules. The temperature during data collection was controlled using a helium cryostat (7 ± 0.2 K). The loadings of CO_2 were performed by volumetric method at ambient temperature, in order to ensure that CO_2 was present in the gas phase when not adsorbed and also to ensure sufficient mobility of CO_2 inside the crystalline structure of MFM-300(V^{III}). NPD data were collected for the bare material, and the material dosed with 1.0 and 2.0 CO_2 per vanadium. The sample was then slowly cooled down to 7 K to ensure CO_2 was completely adsorbed with no condensation in the cell. Sufficient time was allowed to achieve thermal equilibrium before data collection. Rietveld refinements on the NPD patterns of the bare MOF and the samples with various CO_2 loadings were performed using the TOPAS software package. The initial Fourier difference maps were used to find the isosurfaces of the three-dimensional difference scattering-length density distribution for CO_2 molecules. In

this treatment the CO_2 molecules were treated as rigid bodies; we first refined the centers of mass, orientations, and occupancies of the adsorbed CO_2, followed by full profile Rietveld refinement including the positions of metals and linkers, together with their corresponding lattice parameters, resulting in satisfactory R-factors. The final refinements on all the parameters including fractional coordinates, thermal parameters, occupancies for both host lattice and adsorbed CO_2 molecules, and background/profile coefficients yielded very good agreement factors. No restriction of the molecule position was used in the refinement. The total occupancies of CO_2 molecules obtained from the refinement are also in good agreement with the experimental values for the CO_2 loading.

DFT Calculation for INS spectra

Modelling by Density Functional Theory (DFT) of the bare and CO_2-loaded MOFs was performed using the Vienna Ab initio Simulation Package (VASP).[2] The calculation used Projector Augmented Wave (PAW) method[3,4] to describe the effects of core electrons, and Perdew-Burke-Ernzerhof (PBE)[5] implementation of the Generalized Gradient Approximation (GGA) for the exchange-correlation functional. Energy cutoff was 900eV for the plane-wave basis of the valence electrons. The lattice parameters and atomic coordinates determined by NPD in this work were used as the initial structure. Some of the CO_2 sites have partial occupancy, and to account for this properly a supercell calculation would be desirable, but too costly in practice. Instead, a single unit cell was used and the partially occupied sites were modified to be either occupied or unoccupied, according to their local environment and symmetry (there needs to be either a complete CO_2 molecule or no molecule, and the overall probability of being occupied needs to be proportional to the actual occupancy). The total energy tolerance for electronic energy minimization was 10^{-8} eV, and for structure optimization it is 10^{-7} eV. The maximum interatomic force after relaxation was below 0.005 eV/Å. The optB86b-vdW functional[6] for dispersion corrections was applied. The vibrational

eigen-frequencies and modes were then calculated by solving the force constants and dynamical matrix using Phonopy.[7] The aClimax software[8] was used to convert the DFT-calculated phonon results to the simulated INS spectra.

Supplementary References

1. Sheldrick, G. M. Crystal structure refinement with SHELXL. *Acta Crystallogr., C***71**, 3-8 (2015)
2. Kresse, G. & Furthmüller, J. Efficient iterative schemes for ab initio total-energy calculations using a plane-wave basis set. *Phys. Rev. B*, **54**, 11169-11186 (1996).
3. Blochl, P. E. Projector augmented-wave method. *Phys. Rev. B*, **50**, 17953-17979 (1994).
4. Kresse, G. & Joubert, D. From ultrasoft pseudopotentials to the projector augmented-wave method. *Phys. Rev. B*, **59**, 1758-1775 (1999).
5. Perdew, J. P., Burke, K. & Ernzerhof, M. Generalized gradient approximation made simple. *Phys. Rev. Lett.*, **77**, 3865-3868 (1996).
6. Klimeš, J., Bowler, D. R. & Michaelides, A. Chemical accuracy for the van der Waals density functional. *J. Phys.: Cond. Matt*. **22**, 022201 (2010).
7. Togo, A. & Tanaka, I. First principles phonon calculations in materials science. *Scr. Mater*., **108**, 1-5 (2015)
8. Ramirez-Cuesta, A. J. aCLIMAX 4.0.1, The new version of the software for analyzing and interpreting INS spectra. *Comput. Phys. Comm.* **157**, 226-238 (2004).
